# Retrospective study of an incisional hernia after laparoscopic colectomy for colorectal cancer

**DOI:** 10.1186/s12893-023-02229-7

**Published:** 2023-10-16

**Authors:** Toshinori Kobayashi, Hisanori Miki, Nobuyuki Yamamoto, Soushi Hori, Masahiko Hatta, Yuki Hashimoto, Hiromi Mukaide, Makoto Yamasaki, Kentaro Inoue, Mitsugu Sekimoto

**Affiliations:** Department of Surgery, Kansai Medical University, 2-5-1, Shinmachi, Hirakata, Osaka 573-1010 Japan

**Keywords:** Incisional hernia, Hernia size, Laparoscopic colorectal Surgery

## Abstract

**Purpose:**

This study aimed to examine the incidence of incisional hernia (IH) in elective laparoscopic colorectal surgery (LC) using regulated computed tomography (CT) images at intervals every 6 months.

**Methods:**

We retrospectively examined the diagnosis of IH in patients who underwent LC for colorectal cancer at Kansai Medical University Hospital from January 2014 to August 2018. The diagnosis of IH was defined as loss of continuity of the fascia in the axial CT images.

**Results:**

470 patients were included in the analysis. IH was diagnosed in 47 cases at 1 year after LC. The IH size was 7.8 cm^2^ [1.3–55.6]. In total, 38 patients with IH underwent CT examination 6 months after LC, and 37 were already diagnosed with IH. The IH size was 4.1 cm^2^ [0–58.9]. The IH size increased in 17 cases between 6 months and 1 year postoperatively, and in 1 case, a new IH occurred. 47%(18/38) of them continued to grow until 1 year after LC. A multivariate analysis was performed on the risk of IH occurrence. SSI was most significantly associated with IH occurrence (OR:5.28 [2.14–13.05], p = 0.0003).

**Conclusion:**

IH occurred in 10% and 7.9% at 1 year and 6 months after LC. By examining CT images taken for the postoperative surveillance of colorectal cancer, we were able to investigate the occurrence of IH in detail.

**Supplementary Information:**

The online version contains supplementary material available at 10.1186/s12893-023-02229-7.

## Introduction

Incisional hernia (IH) has been reported in 5.2–17% of patients who undergo major abdominal surgery [1–6]. Many studies have reported the causes and preventive measures in which clinical symptoms have mainly been used to diagnose IH [7–15]. However, patients with a small IH are often unaware of their condition, making it difficult to accurately calculate the occurrence rate. It is also difficult to objectively assess the severity of the condition [16]. Some studies have employed imaging modalities, such as computed tomography (CT) and ultrasonography as diagnostic means [16]. However, most studies have included various surgical procedures, and the timing for investigating IH occurrence after surgery has not been unified [13, 17], resulting in inaccuracies in IH analysis [18]. Many Japanese cancer centers and high-volume hospitals have conducted postoperative surveillance of colorectal cancer in accordance with the guidelines established by the Japanese Society for Cancer of the Colon and Rectum [19]. These guidelines recommend performing CT every six months after radical surgery. We believe that these scheduled CT images could be used to conduct a detailed investigation of IHs. This study aimed to examine the incidence of IH in elective laparoscopic colorectal surgery using regulated CT images intervals every 6 months.

## Methods

**Fig. 1:**
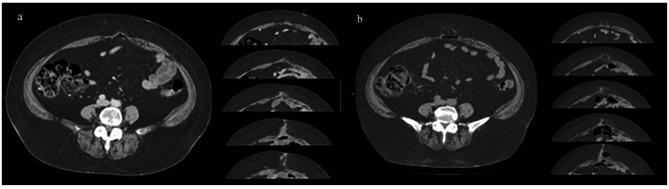
Example of CT images of diagnosed IH occurrence. **a)** Computed tomography (CT) images at 6 months postoperatively. The black line indicates the fascial defect. The length of the horizontal axis was 1.7 cm (minor axis). Six slices of the CT images showed fascial defects; the length of the vertical axis was 3.0 cm (the major axis). The incisional hernia (IH) size was calculated as the ellipse area at 4.0 cm^2^**b)** CT images were taken 1 year postoperatively. The black line indicates the fascial defect. The length of the horizontal axis was 3.4 cm (minor axis). Fifteen slices of the CT images showed fascial defects; the length of the vertical axis was 7.5 cm (the major axis). The size of the IH was calculated as the elliptical area at 20.0 cm^2^

Patients who underwent elective surgery for primary colorectal cancer at Kansai Medical University Hospital between January 2014 and August 2018 were included in the study. However, 2016 was excluded from this study due to the unclear method of closing abdominal fascia. Of the 685 surgeries performed during this period, 126 were performed using an open abdomen method, and 559 were performed laparoscopically. In this study, we analyzed cases of laparoscopic surgery, which is the current standard for colorectal surgery [5]. In our study, all pathological stages were included as oncologic statuses. The site of tumor resection was recorded according to the surgical procedure in the enrolled cases, and the lengths of the tumor specimens were calculated. The details of postoperative adjuvant chemotherapy for stage III colorectal cancer were not collected, as reports indicate that it is unrelated to the long-term prognosis of IH occurrence [20]. We performed midline incision at the umbilicus for specimen extraction because the open method to establish the first port is convenient and safe; it is convenient to extract the specimen by enlarging the incision, depending on the size of the tumor, and laparoscopic procedures were performed via four to five ports. In this study, we examined the occurrence of IH at this incision. Patients were enrolled during the data collection period, and two methods of abdominal fascia closure were used. In the first half of the study period, interrupted sutures using polyglycolic acid (0-Opepolix™; Alfresa Pharma, Osaka, Japan) were used. In the latter period, continuous barbed sutures using polydioxanone, 0-Stratafix™ (Johnson & Johnson, New Brunswick, NJ, USA), were used. Clinical information was collected by referring to medical records. Data on age, sex, body mass index (BMI), underlying conditions, previous surgery, tumor location, tumor diameter, prognostic nutritional index [PNI; calculated as 10 × serum albumin (g/dL) + 0.005 × total lymphocyte count (/mm^3^]) [21], and surgical site infection (SSI) were collected. SSI was defined as a superficial incision site. The diagnosis of IH was defined as loss of continuity of the fascia in the axial CT images [22]. CT images were taken at 5-mm intervals in all cases (Fig. 1). The size of the IH (cm^2^) was defined by measuring the lengths of the horizontal and vertical axes of the defect in the CT images, with the larger axis being the major axis and the smaller axis being the minor axis, calculated based on the area formula of an ellipse (major axis/2 × minor axis/2 × π). This study was approved by the Ethical Review Committee of the Kansai Medical University (approval no. #2,019,210). Informed consent was obtained from the patients using the opt-out method owing to the retrospective design of the study, with no risk to the participants. Information regarding this study, such as the inclusion criteria and the opportunity to opt-out, was provided through the institutional website.

### Statistical analysis

Statistical analyses were performed using JMP Start Statistics version 13.2.0 (Statistical Discovery Software; SAS Institute, Cary, NC, USA). Quantitative data were expressed as medians and ranges. Comparisons between the two groups were made using the Mann–Whitney U test, x^2^ test, and Fisher’s exact test, and *p*-values less than 0.05 were considered statistically significant. Variables with *p*-values of less than 0.05 in the univariate analysis were included in the multivariate analysis. Multivariate analysis was performed using the Cox regression analysis method. The results of the Cox model analysis are reported using odds ratios (ORs) and 95% confidence intervals (CIs). Univariate analyses were performed to identify the risk factors for IH occurrence at 1 year and 6 months postoperatively using the following variables: sex, comorbidities (diabetes and pulmonary disease), previous surgery, SSI, BMI, tumor size, and PNI.

## Results

**Fig. 2:**
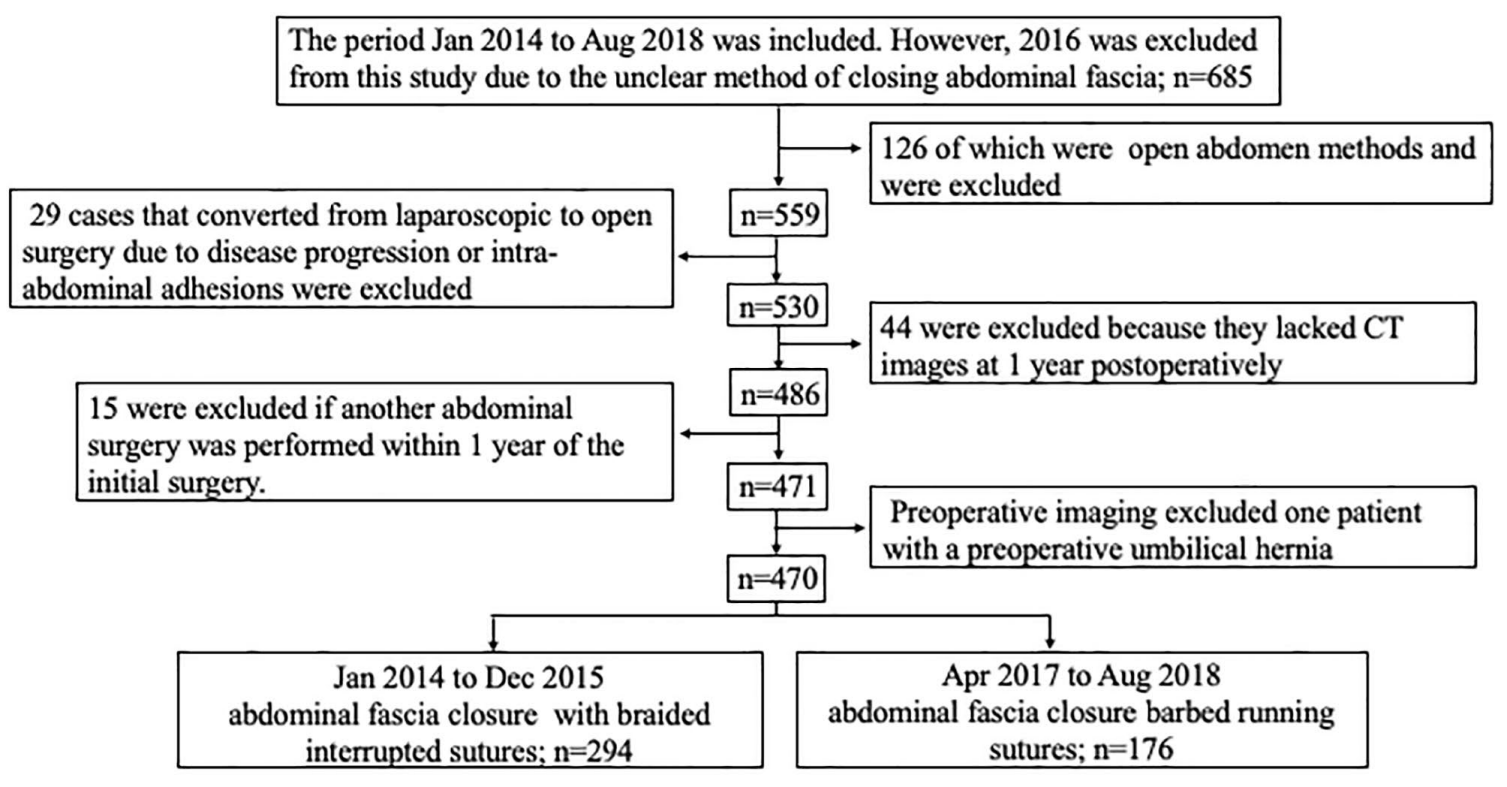
Study flowchart. Data were extracted from 685 cases, 126 of which were open abdomen methods and were excluded from analysis; of the 559 remaining cases, 29 cases that converted from laparoscopic to open surgery due to disease progression or intra-abdominal adhesions were excluded. Of the 530 patients, 44 were excluded because they lacked CT images at 1 year postoperatively. In addition, 15 of the 486 patients were excluded if another abdominal surgery was performed within 1 year of the initial surgery. Preoperative imaging excluded one patient with a preoperative umbilical hernia; ultimately, 470 patients were included in the study. In the first half of the study period, the abdominal fascia was closed with braided interrupted sutures, and in the latter half, it was closed with barbed running sutures

Of the 559 cases, 29 were converted to open abdomen surgery because of disease advancement or intraperitoneal adhesions. CT images were lacking in 44 patients at 1 year postoperatively, and another abdominal operation was performed within 1 year following the initial surgery in 15 patients. One patient had an umbilical hernia prior to the initial surgery. After excluding these cases, 470 patients were included in the analysis. In accordance with institutional procedure changes in the method of abdominal fascia closure over the course of the study, patients in the first period (n = 294) received braided interrupted sutures, and patients in the latter period (n = 176) received barbed running sutures (Fig. 2). The patients’ characteristics are listed in Table 1. The median age was 71 years [39–99], 199 patients (42%) were female, and the median BMI was 22.7 kg/m^2^ [12.4–47.2]. Tumor location consisted of 117 sites on the right side, 126 on the left side, and 227 in the rectum, with a median tumor length of 35 mm [0–167].

**Table 1:** Baseline characteristics of patients undergoing laparoscopic colorectal surgery

Variable	N = 470 (%)
Age, in years, median [IQR^a^]	71 [39–99]
Female sex, n (%)	199 (42)
BMI^b^, kg/m^2^, median [IQR^a^]	22.7 [12.4–47.2]
Diabetes, n (%)	89 (19)
Pulmonary disease, n (%)	35 (7)
Previous surgery	91
Tumor sites: Right/ Left / Rectum	117/ 126/ 227
Tumor length, mm, median [IQR]	35 [0–167]
PNI^c^, median [IQR]	50.1 [23.2–72.2]
SSI^d^, n (%)	25 (5)

^a^IQR, interquartile range; ^b^BMI, body mass index; ^c^PNI, prognostic nutritional index (calculated as 10 × serum albumin [g/dL] + 0.005 × total lymphocyte count [/mm^3^]); ^d^SSI, surgical site infection

**Fig. 3:**
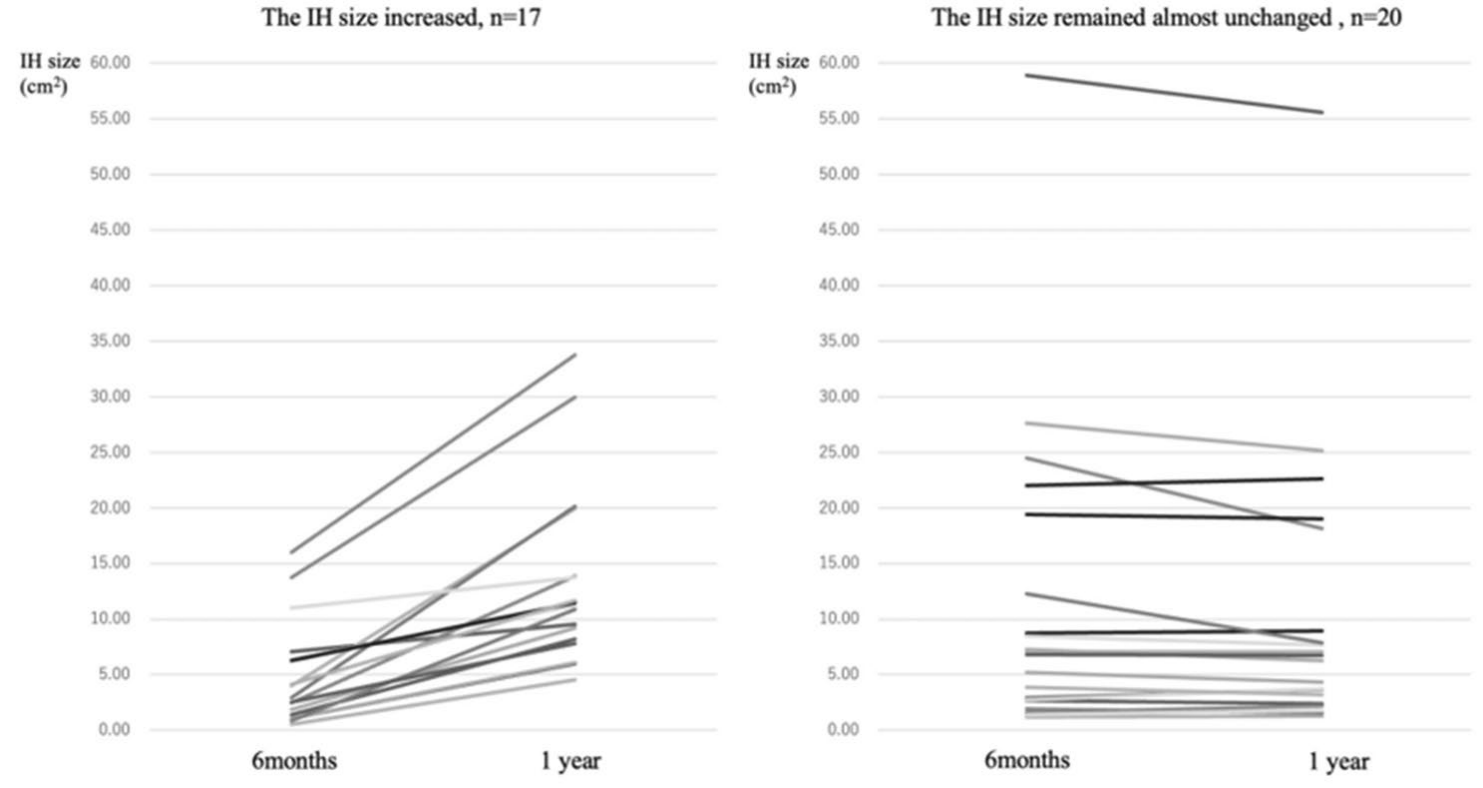
Change diagram in the incisional hernia (IH) size. Among 47 patients with IH, 38 underwent CT examination 6 months after surgery, and IH was detected at that time in 37 cases. The IH size increased in 17 cases between 6 months and 1 year postoperatively as shown in the diagram, and in 1 case, a new IH occurred. However, the IH size was almost unchanged in the remaining 20 cases (52%)

IH was diagnosed in 47 cases at 1 year after surgery. The major axis of the IH was 5.2 cm [2.5–12.5]. The minor axis was 1.8 cm [0.4–5.6], and the IH size was 7.8 cm^2^ [1.3–55.6] (Table 2). In total, 38 patients with IH underwent CT examination 6 months after surgery, and 37 were already diagnosed with IH. The major axis of the IH was 3.0 cm [0–12.5], the minor axis was 1.8 cm [0–6.0], and the IH size was 4.1 cm^2^ [0–58.9] (Table 2). The IH size increased in 17 cases between 6 months and 1 year postoperatively, and in 1 case, a new IH occurred. However, the IH size was almost unchanged in the remaining 20 cases (52%) (Fig. 3).

**Table 2:** The diagnosis and the size of incisional hernia (IH), at 1 year and at 6 months, respectively after laparoscopic colorectal surgery

Variable	N = 470 (%)		
IH, at 1 year	N = 47 (10)	IH, at 6 months	N = 38 (8)
Major axis, cm, median [IQR^a^]	5.2 [2.5–12.5]	Major axis, cm, median [IQR^a^]	3 [0–12.5]
Minor axis, cm, median [IQR^a^]	1.8 [0.36–5.6]	Minor axis, cm, median [IQR^a^]	1.8 [0–6.0]
IH size, cm^2^, median [IQR^a^]	7.8 [1.3–55.6]	IH size, cm^2^, median [IQR^a^]	4.1 [0–58.9]

^a^IQR, interquartile range

Univariate analysis was performed on the risk of IH occurrence at 1 year and 6 months postoperatively. Female sex, BMI, and SSI were significantly associated with IH occurrence (Table 3). Multivariate analysis identified female sex (OR:2.72 [1.41–5.22], p = 0.0027; OR:2.98 [1.43–6.23], p = 0.0035), BMI > 25 (OR:3.10 [1.58–1.34], p = 0.0010; OR:2.58 [1.21–5.52], p = 0.0137), and SSI (OR:5.28 [2.14–13.05], p = 0.0003; OR:5.89 [2.29–15.09], p = 0.0002) as risk factors at 1 year and 6 months, respectively (Table 4).

**Table 3:** Univariate analysis of risk factors for the occurrence of incisional hernia (IH) at 1 year and at 6 months

Variable	Non-IH N = 423	IH at 1 year N = 47	Univariate analysis p-value, at 1 year	IH, at 6 months N = 37	Univariate analysis p-value, at 6 months
Age, median [IQR^a^]	71 [26–99]	67 [41–85]	0.03	67 [41–85]	0.11
Female sex, n (%)	170 (40)	29 (62)	0.005	24 (65)	0.0039
BMI^b^, kg/m^2^, median [IQR^a^]	22.5 [12.4–37.4]	24.6 [18–47.2]	< 0.0001	24.4 [18–47.2]	0.0007
Diabetes, n (%)	81 (19)	8 (17)	0.72	7 (19)	0.99
Pulmonary disease, n (%)	30 (7)	5 (10)	0.38	3 (8)	0.87
Previous surgery, n (%)	83 (20)	8 (17)	0.67	6 (16)	0.61
Tumor size (mm), median [IQR^a^]	35 [0–167]	39 [8–80]	0.45	39 [8–80]	0.46
PNI^c^, median [IQR^a^]	50.2 [23.2–72.2]	48.3 [40.6–65.5]	0.70	50.6 [40.6–65.5]	0.64
SSI^d^, n (%)	17 (4)	9 (19)	< 0.0001	8 (22)	< 0.0001

^a^IQR, interquartile range; ^b^BMI, body mass index; ^c^PNI, prognostic nutritional index (calculated as 10 × serum albumin [g/dL] + 0.005 × total lymphocyte count [/mm^3^]); ^d^SSI, surgical site infection

**Table 4:** Multivariate analysis of risk factors for the occurrence of incisional hernia (IH) at 1 year and at 6 months

Variable	Odds ratio, at 1 year (95% CI^a^)	p-value	Odds ratio, at 6 months (95% CI^a^)	p-value
Female sex	2.72 [1.41–5.22]	0.0027	2.98 [1.43–6.23]	0.0035
BMI^b^, kg/m^2^, > 25	3.10 [1.58–1.34]	0.0010	2.58 [1.21–5.52]	0.0137
SSI^c^	5.28 [2.14–13.05]	0.0003	5.89 [2.29–15.09]	0.0002

^a^CI, confidence interval; ^b^BMI, body mass index; ^c^SSI, surgical site infection;

## Discussion

This study aimed to examine the occurrence of IH in detail using scheduled CT images taken under uniform conditions after laparoscopic colorectal surgery for colorectal cancer, and was the first study to investigate IH size enlargement after the onset of IH in several patients. Our study showed that many IHs occurred very early after surgery at 6 months, with a significant increase in size. However, for more than half of the IH cases, the size of the IH remained almost unchanged. We observed that IH occurred early after surgery in many cases. When IH size was a small stage, early intervention at 6 months postoperatively may allow enhanced treatment of obese patients at high risk of recurrence after IH repair [23].

The incidence of IH is reportedly 9–20% in major abdominal surgery, and in laparoscopic surgery is lower [5, 24]. However, many analyses have been based on clinical symptoms, and some reports have discussed data extracted from registration databases, where the diagnosis was based on various methods [4]. Lee et al. reviewed 17 papers on the occurrence of IH at the umbilical incision following laparoscopic colorectal surgery [6]. Among these, the diagnosis was solely based on physical examination and/or radiological examination in 12, and two did not mention the method used for the diagnosis—only two papers based the diagnosis on CT scans. Deerenberg et al. described that the quality of IH diagnosis based on clinical symptoms was inferior to that based on imaging modalities, such as CT and ultrasonography [17]. Without adequate diagnostic imaging, small IH tend to be missed and underestimated [16]. Furthermore, many reports have not provided details regarding IH occurrence. As mentioned earlier, we performed CT examinations every six months following colorectal cancer surgery. In all cases, CT images were taken at a thickness of 5 mm. We believe that this CT examination enabled precise detection of the IH and determination of the manner and timing of IH development by close evaluation of routine CT images following colorectal cancer surgery.

First, we examined the occurrence of IH 1 year after surgery and found that an IH occurred in as many as 10% of the cases (47/470). This result is almost the same as that previously reported [6]. We diagnosed IH with a loss of continuity of the fascia, even if the defect was only 0.4 cm (minor axis). This strict definition may be responsible for the high incidence of IH in our study. However, as will be described later, some of these small defects significantly increased in size during the previous six months. Thus, we believe that even small defects should be considered as an IH. Among them, three IH cases were symptomatic and were surgically repaired 1 year after the initial surgery. These symptomatic cases had an IH with a size of 22.6 cm^2^–55.6 cm^2^, whereas the median size of all IH cases was 31.1 cm^2^. The IH size increase was not associated necessarily clinical symptoms.

Among 47 patients with IH, 38 underwent CT examination 6 months after surgery, and IH was detected at that time in 37 cases. The IH size was 4.1 cm^2^, signifying that many IHs might occur in the very early period after surgery. In 17 cases, the IH size significantly increased between 6 months and 1 year after surgery; notably, there was only 1 new case of IH during that time.

Furthermore, we analyzed the clinical factors associated with IH. Female sex, BMI, and SSI were evaluated as risk factors. Logistic regression analysis revealed that SSI was the most significant risk factor for IH occurrence. These results are similar to those previously reported [3]. Regarding the method of abdominal fascia closure, the occurrence of IH was not different between the two techniques (p = 0.075). The IH size also did not differ between the two groups (p = 0.42). However, in this study, the method of fascia closure was the only factor associated with IH size growth (braided interrupted sutures vs. continuous barbed running sutures (OR: 20.51 [2.75–446.26], p = 0.0017) (Supplementary Information 1). Interestingly, 6 months after the primary surgery, the median IH size was larger in patients who had undergone surgery with barbed running sutures of 7.0 cm^2^ than in those who had undergone surgery with braided interrupted sutures of 4.0 cm^2^, suggesting that the IH size tends to grow with the braided interrupted closure method between 6 months and 1 year after colorectal surgery (Supplementary Information 1). To our knowledge, no study has examined the factors associated with increasing IH size after IH occurrence. The mechanisms underlying the present supplemental results are unclear; therefore, further long-term follow-up and a search for causes are necessary.

This study had several limitations. First, CT interpretation was performed by the members involved in the research, and not by the radiologist. Although abdominal fascia defect is relatively easy to determine, this may have led to some bias. Claes et al. also suggested that CT examinations performed for colorectal cancer surveillance may detect IH earlier and more frequently [25]. Secondly, as this study was conducted to investigate the occurrence of IH within 1 year of surgery, it is unclear whether an increase in IH size has a relationship with future clinical symptoms. Jensen et al. reported that IH patients who did not undergo repair procedures had reduced long-term quality of life (QOL) in the domains Physical functioning and Role functioning [26]. We might need to observe symptoms of small IH in the long term. Thirdly, as the study was conducted at a single institute, it is unclear whether the findings can be generalized. Fourthly, this study was employed for midline incision because of its simplicity and safety, however, compared to the off-midline and Pfannenstiel incision, Pfannenstiel incision reported good outcomes for preventing IH [27, 28]. Most of them were based on retrospective observational data from clinical findings or hospital databases. Fifthly, this study data was not collected the details of postoperative adjuvant chemotherapy. The adjuvant chemotherapy for colon cancer was recently associated with a high incidence of IH [29], however, many prospective randomized studies evaluating the effect of adjuvant chemotherapy were conducted, and none reported an increased risk of IH by any regimen of chemotherapy [30–32]. So, this study did not analyze the influence of chemotherapy. The relationship between adjuvant chemotherapy and IH should be evaluated in large-scale clinical trials that define methods for diagnosing IH and regimens. Finally, most of the IHs detected in this study were not noted by the radiologist who read the CT images; even cases where the IH increased in size at 6 months were overlooked. In general, treatment for IH is considered after clinical symptoms appear; however, the outcomes are not always favorable. We believe that if IHs are monitored more carefully and treated earlier, treatment results could improve. In Japan, the same surveillance method has been used after colorectal cancer surgery in many hospitals. Therefore, it is possible to investigate IH occurrence and enlargement using uniform criteria throughout the country. We are currently planning a multicenter prospective study to investigate the occurrence and subsequent outcomes of IH.

## Conclusion

In previous reports, IH was evaluated mainly by clinical symptoms, but in this study, using scheduled CT images taken under uniform conditions were used to investigate the occurrence of IH. As a result, we found that CT for postoperative surveillance is effective for early diagnosis of IH. The surveillance examination by CT revealed that IH occurred in about 7.8% of patients six months after surgery. And 47% of them continued to grow until 1 year after surgery. Clinical symptoms were also observed in some cases, but there was no association with IH size. Furthermore, in the future, we would like to examine whether early restoration of IH, such as that found in this study, is clinically useful. Many surgeries for malignancies involve postoperative surveillance using CT. If more physicians realize that these are useful for early detection IH, treatment of IH may change.

### Electronic supplementary material

Below is the link to the electronic supplementary material.

Supplementary Material 1

## Data Availability

The datasets generated and analyzed during the current study are available from the corresponding author on reasonable request.
