# Supplementary material for: Retrospective study of an incisional hernia after laparoscopic colectomy for colorectal cancer
Source: BMC Surg. 2023 Oct 16;23:314. doi: 10.1186/s12893-023-02229-7 (PMC10580507; doi:10.1186/s12893-023-02229-7)
Supplement: Supplementary file 1 — Supplementary Material 1 [file 12893_2023_2229_MOESM1_ESM.docx]

Supplementary Information 1. The Factors contributing to increase of incisional hernia (IH) size by fascia closer methods

| **Variable** |  | **Braided interrupted** | **Barbed**  **running** | **p value** |
| --- | --- | --- | --- | --- |
| **IH, at 1 year** | N=47 | N=35 | N=12 | 0.075 |
| Major axis (cm) / median [IQR^a^] | 5.2 [2.5–12.5] | 6 [2.5–12.5] | 5 [2.5–10] | 0.55 |
| Minor axis (cm) | 1.8 [0.36–5.6] | 1.9 [0.4–5.7] | 1.6 [0.8–4.3] | 0.44 |
| IH size (cm^2^) | 7.8 [1.3–55.6] | 9 [1.3–55.6] | 6.55 [1.5–33.8] | 0.42 |
| **IH, at 6 months** | N=38 | N=28 | N=10 | 0.17 |
| Major axis (cm) / median [IQR^a^] | 3 [0–12.5] | 3 [0–12.5] | 3.5 [2–8] | 0.13 |
| Minor axis (cm) | 1.8 [0–6.0] | 1.7 [0–6.0] | 2.55 [0.9–4.4] | 0.32 |
| IH size (cm^2^) | 4.1 [0–58.9] | 4 [0–58.9] | 7 [1.4–27.6] | 0.21 |

| **N=38** | **Univariate analysis**  **p-value** | **Multivariate analysis**  **p-value** | **Odds ratio** |
| --- | --- | --- | --- |
| Braided interrupted/Barbed running | 0.0166 | 0.0211 | 16.41 [1.52–176.98] |

^a^BMI, body mass index; ^b^SSI, surgical site infection
